# A Dietary Pattern Derived by Reduced Rank Regression is Associated with Type 2 Diabetes in An Urban Ghanaian Population

**DOI:** 10.3390/nu7075233

**Published:** 2015-07-07

**Authors:** Laura K. Frank, Franziska Jannasch, Janine Kröger, George Bedu-Addo, Frank P. Mockenhaupt, Matthias B. Schulze, Ina Danquah

**Affiliations:** 1 Department of Molecular Epidemiology, German Institute of Human Nutrition Potsdam-Rehbruecke, Arthur-Scheunert-Allee 114-116, 14558 Nuthetal, Germany; E-Mails: laura.frank@dife.de (L.K.F.); franziska.jannasch@dife.de (F.J.); janine.kroeger@dife.de (J.K.); mschulze@dife.de (M.B.S.); 2 Komfo Anokye Teaching Hospital, School of Medical Sciences, Kwame Nkrumah University of Science and Technology, Kumasi, Ghana; E-Mail: gbeduaddo@gmail.com; 3 Institute of Tropical Medicine and International Health, Charité–University Medicine Berlin, Campus Virchow-Klinikum, Augustenburger Platz 1, 13353 Berlin, Germany; E-Mail: frank.mockenhaupt@charite.de

**Keywords:** adiponectin, biomarker, dietary pattern, HDL-cholesterol, reduced rank regression, sub-Saharan Africa, triglyceride, type 2 diabetes

## Abstract

Reduced rank regression (RRR) is an innovative technique to establish dietary patterns related to biochemical risk factors for type 2 diabetes, but has not been applied in sub-Saharan Africa. In a hospital-based case-control study for type 2 diabetes in Kumasi (diabetes cases, 538; controls, 668) dietary intake was assessed by a specific food frequency questionnaire. After random split of our study population, we derived a dietary pattern in the training set using RRR with adiponectin, HDL-cholesterol and triglycerides as responses and 35 food items as predictors. This pattern score was applied to the validation set, and its association with type 2 diabetes was examined by logistic regression. The dietary pattern was characterized by a high consumption of plantain, cassava, and garden egg, and a low intake of rice, juice, vegetable oil, eggs, chocolate drink, sweets, and red meat; the score correlated positively with serum triglycerides and negatively with adiponectin. The multivariate-adjusted odds ratio of type 2 diabetes for the highest quintile compared to the lowest was 4.43 (95% confidence interval: 1.87–10.50, *p* for trend < 0.001). The identified dietary pattern increases the odds of type 2 diabetes in urban Ghanaians, which is mainly attributed to increased serum triglycerides.

## 1. Introduction

Type 2 diabetes is one of the major public health problems in sub-Saharan Africa (SSA) [1,2]. The prevalence rates are rising rapidly. Obesity, physical inactivity and urbanization substantially contribute to this development [3]. Nutritional behavior as a potentially modifiable risk factor is thought to have an important influence on the development of diabetes, but has only been insufficiently examined as a contributing factor for type 2 diabetes in SSA. The use of dietary patterns in nutritional epidemiology has increased in the past [4]. Dietary patterns reflect different combinations of food intake and allow the assessment of the overall diet instead of single nutrients or food items. Two general approaches facilitate the identification of dietary patterns: the hypothesis-oriented approach uses prior knowledge such as diet-quality scores based on diet recommendations or guidelines and the exploratory approach, such as factor and cluster analysis that are entirely empirical data-driven methods [5,6]. Reduced rank regression (RRR) has been proposed as a new dimension-reduction technique, which combines both approaches [7]. It combines the dietary information with prior scientific knowledge of the pathway from diet to disease. RRR determines linear combinations of predictor variables (e.g., food groups) by maximizing the explained variation in a set of response variables (e.g., nutrients [7,8,9] or biomarkers [10]) that are presumed to be related to the disease of interest. Previous studies reported strong associations between dietary patterns and type 2 diabetes by using RRR [7,11,12,13,14]. These dietary patterns were derived in Western populations but not in SSA. On the background of scarce epidemiologic data regarding nutritional behavior and type 2 diabetes in SSA, RRR appears to be a promising tool to characterize diet–disease relationships in this region. Yet, this approach remains to be attempted. Therefore, the aims of this study were, first, to identify a dietary pattern associated with serum concentrations of diabetes-related biomarkers (adiponectin, HDL-cholesterol and triglycerides) by using RRR in a training set of an urban Ghanaian study population (*n* = 603); second, to apply this dietary pattern score to a validation set of the same study population (*n* = 603); and third, to evaluate associations of the identified dietary pattern with type 2 diabetes in both sets of the urban Ghanaian study population.

## 2. Materials and Methods

### 2.1. Study Population

The Kumasi Diabetes and Hypertension (KDH) Study is an unmatched case-control study that was conducted at Komfo Anokye Teaching Hospital (KATH) in Kumasi, Ghana from August 2007 through June 2008. The primary aim was to identify risk factors for type 2 diabetes (and hypertension). The detailed description of the recruitment procedures and the characteristics of the study population are provided elsewhere [15]. Briefly, cases were recruited from the diabetes center (*n* = 495) and the hypertension clinic (*n* = 451). They encouraged their friends, neighbors and community members (*n* = 222) to participate in the study as potential controls. Further preliminary controls came from the outpatient department (*n* = 150) and hospital staff (*n* = 148). Every participant underwent a clinical examination and a personal interview on the socio-demographic background, the medical history, and the economic status. Fasting venous blood samples were drawn. For study purposes, type 2 diabetes was defined as having a fasting plasma glucose (FPG) ≥7 mmol/L and/or as being on documented anti-diabetic medication [16]. We refrained from HbA1c-based definition due to potential misclassification in individuals with hemoglobinopathies and malaria infection. Controls were defined as participants without diabetes. Of the 1466 participants included in the study, 260 were excluded from the present analysis due to missing information on nutrition (141), anthropometry (39), socio-economic status (SES, 31), genetic polymorphisms (34), and biomarkers (15). Hence, this analysis comprised 1206 individuals (668 controls, 538 diabetes cases).

All participants provided written informed consent; the study protocol was reviewed and approved by the Ethics Committee of the School of Medical Sciences, University of Science and Technology, Kumasi.

### 2.2. Dietary Assessment

The methods of the nutritional assessment have previously been described in detail [17]. Briefly, a locally specific food frequency questionnaire (FFQ) was applied to all participants in face-to-face interviews by trained nurses who speak the local language. This questionnaire assessed the usual weekly intake frequency of 51 food items in 10 food categories consumed during the past 12 months. Information about food groups and food categories are presented in Table S2. The food categories were starchy roots and plantain; cereals and cereal products; animal products; legumes, nuts and oilseeds; fruits; vegetables; fats and oils; salt and spices; sweets; and liquids. No portion sizes were available. Thus, the FFQ covered intake frequencies, but not quantities of food consumption. The frequency of intake was measured using six categories: never, seldom (<time per week), 1–2 times per week, 3–4 times per week, 5–6 times per week, and daily. This FFQ has not been validated yet.

### 2.3. Covariate Assessment

Waist circumference (cm), hip circumference (cm), weight (kg) and height (cm) were measured (all devices Seca, Germany) by trained study personnel. BMI was calculated as weight/(height)^2^ (kg/m^2^), and waist-to-hip ratio (WHR) was computed as waist circumference/hip circumference. Socio-demographic data and the medical history comprised age, sex, own and family history of diabetes (yes, no), and smoking behavior (never, quit, current). SES data included information about education (none, primary, secondary, tertiary, other), literacy (able or unable to read and write), occupation (subsistence farmer, commercial farmer, casual laborer, artisan, trader, businessman/woman, public servant, unemployed, other), number of people living in the household and household assets: electricity, pipe water, fan, fridge, cupboard, radio, TV, bicycle, motor bike, car, truck and tractor and cattle (yes/no). These variables were used to construct a SES sum score. It ranges from 0 to 12 points and comprises the domains education, occupation and income. The exact procedure of the construction is depicted in Figure S1. Self-reported physical activity comprised work-related, transportation-related and leisure-time physical activity. Duration (min/week) and type (*i.e.*, intensity) of physical activity were translated into daily energy expenditure (kcal/day) as the sum of metabolic equivalents corresponding to activity intensity (mL/kg/min) × body weight (kg) × duration (min) [18].

### 2.4. Laboratory Procedures

Each participant provided a fasting blood sample (fluoride plasma, tubes cooled at +4 °C). FPG was measured photometrically (Glucose 201+ Analyzer, HemoCue, Ångelholm, Sweden) and is presented as plasma equivalents. Serum triglycerides and HDL-cholesterol were measured by colorimetric assays (ABX Pentra400, Horiba Medical, Reichenbach, Germany). The inter-assay CVs were 4.5% and 1.8%, respectively. The total adiponectin concentration was measured using a commercially available ELISA with intra- and inter-assay CVs of 4.9% and 6.7% (BioVendor, Heidelberg, Germany).

### 2.5. Statistical Analysis

#### 2.5.1. Descriptive Analysis

For the comparison of baseline characteristics between diabetes cases and controls, the non-parametric Mann–Whitney U test was used for continuous and χ^2^-test for categorical variables. Spearman correlations were used to assess the relationship between biomarkers among the control group.

#### 2.5.2. Reduced Rank Regression

We applied RRR to derive a dietary pattern predictive of the diabetes status. This method has been described in detail by Hoffmann *et al.* [7], including SAS code and its application in nutritional epidemiology, and has been applied in recent studies of dietary patterns and chronic diseases [10]. The directed acyclic graph in Figure S2 describes the conceptual framework of the present RRR. Briefly, RRR determines linear combinations of predictor variables (e.g., food groups) that explain as much as possible variation in the response variables (e.g., biomarkers). It is based on the assumption that canonical correlations between predictor and response variables are negligibly small. In this analysis, we used 35 food items as predictor variables and serum concentrations of HDL-cholesterol, log-transformed adiponectin and log-transformed triglycerides as response variables. Thus, dietary patterns that explained a maximum of variation in these biomarkers were identified. The response variables adiponectin, HDL-cholesterol and triglycerides were chosen based on their established relationship with type 2 diabetes [19,20,21]. Since we are lacking an external validation population to test the robustness of our results, we randomly split our study population (*n* = 1206) into two subsamples (each *n* = 603) and performed an internal validation. We calculated the dietary pattern score in one subsample (training set) and applied this pattern score to the other (validation set). The dietary pattern score was calculated as the sum of z-standardized intakes (mean = 0, standard deviation = 1) of 35 food items multiplied by an individual weight. For the calculation of the dietary pattern score, all 35 food items were subject to analysis. However, henceforth, we will focus the description of the dietary pattern on those ten food items with the highest factor loadings. These were considered to be the main contributors to the score. Each participant received a factor score for the identified dietary pattern. These scores were used to rank participants according to the degree to which they conformed to the dietary pattern.

Based on the distribution among the control group, quintiles of the dietary pattern score were constructed. Socio-demographic and anthropometric characteristics, the SES sum score, biomarker concentrations and frequencies of food intake were calculated across the quintiles of the dietary pattern score among the control group. The differences among categorical variables (χ^2^-test) and linear trends among continuous parameters (trend test) were assessed.

#### 2.5.3. Dietary Pattern and Type 2 Diabetes

Logistic regression analysis was applied to evaluate the associations between the dietary pattern and type 2 diabetes. Odds ratios (OR) and 95% confidence intervals (CI) were calculated across the quintiles and per 1 SD of the dietary pattern score. The significance of a linear trend across the categories was tested by assigning each control the median of a category and by modeling this value as a continuous variable. For type 2 diabetes, as a rare disease with long latency, we applied the proportional odds assumption in logistic regression models and based our adjustments on previous findings. Initially we adjusted for age and sex (Model 1). Then, we further adjusted for diabetes family history (yes or no), SES sum score, smoking status (never/current or ex-smoker) and energy expenditure (kcal/day) (Model 2), and finally we adjusted for BMI and WHR (Model 3).

#### 2.5.4. Sensitivity Analyses

We tested interactions of the association between the dietary pattern and type 2 diabetes with sex, general obesity (BMI ≥ 30 kg/m^2^) or central adiposity (waist circumference ≥ 102 cm (men), ≥88 cm (women)) by performing stratified analyses and we evaluated the significance of cross-product terms. We also examined the sensitivity of our results and excluded participants with lipid-lowering and anti-inflammatory drug intake. We assessed the association between biomarkers of glucose metabolism (FPG and HOMA-IR) and the dietary pattern score across quintiles among the controls.

Finally, we simplified our dietary pattern score in the total study population by summing up the unweighted standardized intake of the food items with the ten highest factor loadings. To assess the importance of individual food components of the dietary pattern score for type 2 diabetes, we sequentially subtracted each component from the simplified dietary pattern score. We calculated the change in estimate (CIE) as the difference between the ORs divided by the OR from the simplified score multiplied by 100.

We defined a two-sided p-value of <0.05 as statistical significance. All statistical analyses were performed using SAS statistical software (version 9.4, SAS Institute, Cary, NC, USA).

## 3. Results

### 3.1. Study Population

The characteristics of the total study population (*n* = 1206) are presented in Table 1. Compared to the controls, the diabetes cases were on average older, had higher WHR and were of lower SES. In addition, diabetes cases had more often diabetes in their family, tended to smoke more often, and had higher intake of anti-inflammatory drugs and energy expenditure than controls. Biomarker concentrations differed significantly between diabetes cases and controls, with lower values of adiponectin and HDL-cholesterol, but higher values of serum triglycerides, FPG and HOMA-IR in the diabetes group.

Among the control group, adiponectin correlated positively with HDL-cholesterol (*r* = 0.16, *p* < 0.001) and negatively with triglycerides (*r* = −0.17, *p* < 0.001). HDL-cholesterol and triglycerides were weakly correlated with each other (*r* = 0.08, *p* = 0.03).

**Table 1:** Characteristics and biomarkers of 1206 urban Ghanaians of the Kumasi Diabetes and Hypertension Study.

Characteristics	Controls ( *n* = 668)	Diabetes Cases ( *n* = 538)
Sex (female)	516 (77.3)	396 (73.6)
Age (years)	46.8 ± 15.7	54.7 ± 13.4 *
BMI (kg/m^2^)	25.8 ± 5.4	25.7 ± 4.9
Waist-to-hip ratio	0.86 ± 0.08	0.91 ± 0.07 *
Socio-economic status sum score		
Very low (0–4 points)	118 (17.7)	200 (37.2) *
Low (5–8 points)	355 (53.1)	278 (51.7)
Moderate (9–10 points)	195 (29.2)	60 (11.2)
Family history of diabetes (yes)	167 (25.0)	314 (58.4) *
Hypertension (yes)	344 (51.5)	332 (61.7) *
Smoking (ever)	27 (4.0)	40 (7.4) *
Lipid-lowering drug intake	12 (1.8)	16 (3.0)
Anti-inflammatory drug intake	6 (0.9)	18 (3.4) *
Energy expenditure (kcal/day)	1214 (848–1630)	1408 (815–1996) *
Biomarkers		
Adiponectin (mg/mL)	8.63 (6.50–11.63)	7.42 (5.36–9.98) *
HDL-cholesterol (mmol/L)	1.37 (1.13–1.62)	1.27 (1.04–1.54) *
Triglycerides (mmol/L)	1.19 (0.87–1.64)	1.36 (1.02–1.87) *
Fasting plasma glucose (mmol/L)	4.40 (4.10–4.90)	6.90 (5.30–10.30) *
HOMA-IR	1.37 (0.85–2.13)	2.00 (1.17–3.40) *

Values are expressed as mean ± standard deviation, participant number (%) or median (interquartile range); * *p*-value ≤ 0.05.

### 3.2. Dietary Pattern and Biomarkers

After a random split of our study population into two sets of equal size (each *n* = 603) and with an equal ratio of diabetes cases and controls, dietary pattern scores were derived by RRR in the training set. The intake frequencies of 35 food items were used as predictors and concentrations of adiponectin, HDL-cholesterol and triglycerides as responses; we obtained three scores. The first dietary pattern score explained 6.8% of the total variation in foods and 3.9% of the total variation in all three biomarkers and was largely driven by the explained variation in triglycerides (9.9%) and only marginally by HDL cholesterol (1.9%) and adiponectin (0.03%). The second and third RRR score explained 2.2% and 1.7% of the total biomarker variation, respectively. These were not biologically plausible. Therefore, we did not consider them for further analyses. In this training set, the identified dietary pattern was characterized by a high consumption of plantain, cassava and garden egg and a low intake of rice, juice, vegetable oil, eggs, milo (chocolate drink), sweets and red meat (Table 2).

The characteristics and biomarker concentrations across dietary pattern quintiles among the controls in the training set are shown in Table S1. Participants with higher dietary pattern score were older, heavier, of lower SES, had lower adiponectin and HDL-cholesterol, but they showed higher triglyceride concentrations.

In the next step, the identified dietary pattern score was applied to the validation set of our study population. In this set, the characteristics and distributions of biomarkers across quintiles of the pattern score among the controls were similar to those in the training set (Table 3). However, no linear trend was observed for HDL-cholesterol. The median intakes of the food items that contributed most to the dietary pattern varied across the score. For instance, participants in the highest quintile consumed plantain five-times more frequently than those in the lowest quintile. In contrast, the rice intake was five-times lower (Table 3).

**Table 2:** Percentage of food variation explained by first dietary pattern score and factor loadings of all 35 food items derived by reduced rank regression in the training set (*n* = 603).

Food Item	Explained Variation (%)	Factor Loading ^1^
Plantain	23.6	0.31
Cassava	23.0	0.31
Garden egg	16.0	0.26
Rice	24.0	−0.32
Juice	21.7	−0.30
Vegetable oil	19.7	−0.29
Eggs	15.2	−0.25
Milo (chocolate drink)	13.3	−0.24
Sweets	11.8	−0.22
Red meat	11.2	−0.22
Groundnut	9.70	−0.20
Soft drinks	9.00	−0.19
Margarine	7.46	−0.18
Milk	6.87	−0.17
Fruits	5.37	−0.15
Carrot	3.75	−0.13
Beans	3.29	−0.12
Lettuce	2.23	−0.10
Cocoyam	2.61	0.10
Cucumber	1.70	−0.08
Millet	1.44	−0.08
Yam	1.03	−0.07
Green leaves	1.15	0.07
Coffee	0.85	−0.06
Palm oil	0.75	−0.06
Okro	0.18	0.03
Maize (banku)	0.26	0.03
Crab	0.23	0.03
Poultry	0.18	−0.03
Porridge	0.13	0.02
Alcoholic drinks	0.07	0.02
Sweet potato	0.13	−0.02
Agushie (pumpkin seeds)	0.02	0.01
Bread	0.02	−0.01
Fish	0.005	−0.004

^1^ Factor loadings are correlations between food items and the dietary pattern score.

### 3.3. Dietary Pattern and Type 2 Diabetes

The associations with type 2 diabetes for quintiles and per 1 SD of the dietary pattern score for the training and the validation set are presented in Table 4. A higher dietary pattern score increased the odds for type 2 diabetes in both sets. In the validation set, the OR for type 2 diabetes in the highest quintile compared to the lowest was 4.43 (95% CI: 1.87–10.50), *p* for trend < 0.001 in the fully adjusted model. In the training set, this figure was 4.57 (95% CI: 2.14–9.76), *p* for trend < 0.001.

We conducted several sensitivity analyses to assess the robustness of our results. In the validation set, we examined potential modifications of the risk estimates by sex, general obesity or central adiposity. In the fully adjusted model, the associations between the dietary pattern and type 2 diabetes were consistent for men and women (men (*n* = 159): OR = 1.52 (0.99–2.34), women (*n* = 444): OR = 1.60 (1.20–2.12), *p* for interaction = 0.96), for BMI categories (<30.0 kg/m^2^ (*n* = 487): OR = 1.35 (1.05–1.75), ≥30.0 kg/m^2^ (*n* = 116): OR = 2.60 (1.36–4.97), *p* for interaction = 0.76) and for categories of waist circumference (<102 cm (men), <88 cm (women) (*n* = 347): OR = 1.31 (0.97–1.78), ≥102/88 cm (*n* = 256): OR = 1.83 (1.26–2.67), *p* for interaction = 0.56).

Additionally, we repeated the RRR for the identification of the dietary pattern after excluding participants with lipid-lowering (2.3%) or anti-inflammatory drug intake (2.0%). The results were virtually identical (data not shown).

We appreciated the possibility that individuals with type 2 diabetes likely present with abnormal serum concentrations of adiponectin, HDL-cholesterol and triglycerides, and that this might have distorted our findings. Hence, we investigated whether the dietary pattern score was associated with biomarkers of glucose metabolism among apparently healthy controls (*n* = 343) in the validation set. Fasting plasma glucose tended to increase across quintiles of the pattern score (mean ± sd: 4.40 ± 0.67, 4.64 ± 0.76, 4.78 ± 0.72, 4.55 ± 0.70 and 4.64 ± 0.61 mmol/L, *p* for trend = 0.13). The respective values for HOMA-IR were: median (interquartile range): 1.33 (0.96–2.15), 1.64 (0.99–2.51), 1.63 (1.03–2.51), 1.24 (0.82–1.77) and 1.38 (0.87–2.57), *p* for trend = 0.51.

Finally, we assessed the importance of individual food components for type 2 diabetes by sequential removal of each component from the simplified dietary pattern score (Table 5). This analysis was conducted in the total study population, because the associations between the dietary pattern score and type 2 diabetes were similar in the training and the validation set. The exclusion of milo (chocolate drink), juice, plantain, sweets, garden egg, red meat and rice attenuated the OR for type 2 diabetes, whereby the removal of cassava, which was positively associated with the dietary pattern score, increased the association with type 2 diabetes.

**Table 3:** Characteristics and biomarkers by quintiles of dietary pattern score among 343 controls in the validation set.

	Quintile of the Dietary Pattern Score					
**Characteristics**	**1**	**2**	**3**	**4**	**5**	***p* for trend**
*n*	68	70	68	69	68	
Sex (female)	51 (75.0)	53 (75.7)	49 (72.1)	54 (78.3)	54 (79.4)	0.87
Age (years)	32.5 ± 13.4	40.8 ± 13.5	46.6 ± 14.0	53.0 ± 11.8	54.7 ± 14.9	<0.001
BMI (kg/m^2^)	24.0 ± 4.8	25.8 ± 4.7	27.1 ± 6.4	26.4 ± 6.4	27.0 ± 5.8	0.002
WHR	0.82 ± 0.08	0.85 ± 0.07	0.87 ± 0.06	0.87 ± 0.08	0.89 ± 0.08	<0.001
very low SES	3 (4.4)	8 (11.4)	5 (7.4)	21 (30.4)	16 (23.5)	<0.001
Family history of diabetes	18 (26.5)	20 (28.6)	21 (30.9)	18 (26.1)	14 (20.6)	0.73
Smoking (ever)	5 (7.4)	4 (5.7)	3 (4.4)	1 (1.5)	3 (4.4)	0.57
Energy expenditure (kcal/day)	1177 (901–1593)	1289 (949–1731)	1329 (962–1729)	1222 (1015–1687)	1245 (712–1786)	0.92
Biomarkers						
Adiponectin (mg/mL)	9.41 (6.34–11.94)	8.27 (5.86–10.57)	7.89 (5.93–11.93)	8.88 (6.79–12.53)	8.73 (6.52–12.33)	0.19
HDL-cholesterol (mmol/L)	1.37 (1.11–1.62)	1.43 (1.30–1.69)	1.35 (1.19–1.60)	1.36 (1.16–1.53)	1.38 (1.14–1.66)	0.64
Triglycerides (mmol/L)	0.97 (0.69–1.23)	1.26 (0.86–1.63)	1.10 (0.85–1.56)	1.32 (0.97–1.89)	1.48 (0.99–1.86)	<0.001
Food intake (times/week) ^1^						
positive association						
Plantain	1.5 (0.5–3.5)	1.5 (1.5–3.5)	3.5 (1.5–5.5)	5.5 (3.5–7.0)	7.0 (4.5–7.0)	<0.001
Cassava	1.5 (0.5–1.5)	1.5 (1.5–3.5)	1.5 (1.5–3.5)	3.5 (1.5–3.5)	7.0 (3.5–7.0)	<0.001
Garden egg	3.5 (1.5–7.0)	3.5 (1.5–7.0)	5.5 (2.5–7.0)	7.0 (3.5–7.0)	7.0 (7.0–7.0)	<0.001
inverse association						
Rice	7.0 (5.5–7.0)	7.0 (3.5–7.0)	3.5 (3.5–7.0)	3.5 (1.5–5.5)	1.5 (0.5–3.5)	<0.001
Juice	1.5 (0.5–5.5)	1.0 (0.5–3.5)	0.5 (0–1.5)	0 (0–0.5)	0 (0–0.5)	<0.001
Vegetable oil	3.5 (1.5–7.0)	3.5 (1.5–5.5)	3.5 (1.5–3.5)	1.5 (0.5–3.5)	1.5 (0.5–3.5)	<0.001
Eggs	2.5 (1.5–3.5)	1.5 (0.5–1.5)	0.5 (0.5–1.5)	0.5 (0.5–1.5)	0.5 (0–0.5)	<0.001
Milo (chocolate drink)	3.5 (1.5–5.5)	1.5 (0.5–3.5)	1.5 (0.5–3.5)	0.5 (0–3.5)	0.5 (0–1.5)	<0.001
Sweets	0.5 (0.5–1.5)	0.5 (0.5–1.5)	0.5 (0–1.5)	0.5 (0–0.5)	0 (0–0.5)	<0.001
Red meat	3.5 (1.5–7.0)	1.5 (1.5–3.5)	1.5 (0.5–3.5)	0.5 (0.5–1.5)	0.5 (0.5–3.5)	<0.001

Values are expressed as mean ± standard deviation, participant number (%) or median (interquartile range). ^1^ We included 35 food items in our analysis, the ten food items that loaded highest on the dietary pattern derived by reduced rank regression are presented here.

**Table 4:** Odds Ratios (OR) and 95% confidence intervals for type 2 diabetes by quintiles and per 1 SD of the dietary pattern score.

	Odds Ratios (95% confidence intervals) for Quintiles					*p* for trend	OR per 1-score SD
	Quintile 1	Quintile 2	Quintile 3	Quintile 4	Quintile 5		
Training set							
No. of cases/controls	17/65	33/65	58/65	76/65	94/65		
Model 1	1.00 (ref.)	1.69 (0.85–3.37)	2.78(1.43–5.38)	3.44 (1.78–6.65)	4.05 (2.09–7.86)	<0.001	1.55 (1.27–1.88)
Model 2	1.00 (ref.)	1.98 (0.93–4.22)	3.42 (1.65–7.12)	3.84 (1.86–7.95)	5.04 (2.42–10.48)	<0.001	1.59 (1.28–1.96)
Model 3	1.00 (ref.)	1.88 (0.86–4.09)	2.95 (1.39–6.29)	3.28 (1.55–6.94)	4.57 (2.14–9.76)	<0.001	1.52 (1.22–1.89)
Validation set							
No. of cases/controls	10/69	32/70	49/70	64/68	105/70		
Model 1	1.00 (ref.)	2.50 (1.12–5.57)	3.15 (1.43–6.93)	3.76 (1.72–8.24)	6.08 (2.81–13.16)	<0.001	1.74 (1.42–2.13)
Model 2	1.00 (ref.)	2.25 (0.95–5.36)	2.86 (1.21–6.75)	3.04 (1.30–7.11)	5.04 (2.19–11.60)	<0.001	1.60 (1.28–2.00)
Model 3	1.00 (ref.)	2.26 (0.92–5.54)	2.81 (1.15–6.84)	3.20 (1.33–7.70)	4.43 (1.87–10.50)	<0.001	1.52 (1.20–1.92)

Model 1: adjusted for age (years) and sex; Model 2: adjusted for age (years), sex, diabetes family history (yes *vs.* no), SES sum score (metric: 0–12 points), smoking status (ever *vs.* never) and energy expenditure (kcal/d); Model 3: adjusted for age (years), sex, diabetes family history (yes *vs.* no), SES sum score (metric: 0–12 points), smoking status (ever *vs.* never) and energy expenditure (kcal/d), BMI (kg/m²) and waist-to-hip ratio.

**Table 5:** Importance of individual components of the simplified dietary pattern score among the total study population (*n* = 1206).

Dietary Variable	OR per 1SD Score	CIE (%) ^2^
Simplified dietary pattern score ^1^	2.17 (1.80–2.62)	
Simplified dietary pattern score without milo (chocolate drink)	1.74 (1.47–2.07)	−19.8
Simplified dietary pattern score without juice	1.95 (1.63–2.32)	−10.1
Simplified dietary pattern score without plantain	2.02 (1.69–2.43)	−6.9
Simplified dietary pattern score without sweets	2.05 (1.71–2.46)	−5.5
Simplified dietary pattern score without garden egg	2.10 (1.74–2.52)	−5.1
Simplified dietary pattern score without red meat	2.11 (1.76–2.53)	−2.8
Simplified dietary pattern score without rice	2.12 (1.76–2.55)	−2.3
Simplified dietary pattern score without vegetable oil	2.17 (1.80–2.61)	-
Simplified dietary pattern score without eggs	2.25 (1.87–2.70)	+3.7
Simplified dietary pattern score without cassava	2.74 (2.25–3.35)	+26.3

^1^ Simplified dietary pattern score: sum of unweighted standardized intake of 10 food items, which were highly loaded on the dietary pattern (plantain + cassava + garden egg – milo (chocolate drink) – red meat – juice – rice – sweets – eggs – vegetable oil); ^2^ CIE: Change in estimate: difference between ORs divided by OR of simplified dietary pattern score and multiplied by 100 (%); Model 3 adjustments were applied: adjusted for age, sex, diabetes family history, SES sum score, energy expenditure, BMI and waist-to-hip ratio.

## 4. Discussion

Due to rising prevalence rates of type 2 diabetes in SSA, it is necessary to clarify the potential importance of nutritional behavior in this region. Studies from SSA, which investigate the association between dietary patterns and type 2 diabetes are scarce. This led us to examine dietary patterns derived by RRR and their associations with type 2 diabetes in an urban Ghanaian population. In a training set, we identified a dietary pattern that was characterized by a high consumption of traditional foods and a low intake of purchased items. Participants with a higher dietary pattern score had higher concentrations of triglycerides and lower concentrations of adiponectin; the pattern score significantly increased the odds for type 2 diabetes. We verified these findings in an internal validation set of this Ghanaian study population.

The results of the present study confirm previous findings of the KDH Study. The food items that were positively associated with the dietary pattern score in the RRR (plantain and garden egg) were also included in the “traditional” dietary pattern identified by an exploratory factor analysis [17]. This “traditional” dietary pattern was characterized by high intakes of plantain, green leafy vegetables, beans, garden egg, fruits, fish, fermented maize products and palm oil and was positively associated with type 2 diabetes. Those food items that were inversely associated with the pattern score in RRR (rice, juice, eggs, milo (chocolate drink), sweets and red meat) were also included in the “purchase” pattern identified by the factor analysis. This “purchase” pattern was characterized by a high consumption of sweets, rice, red meat, poultry, eggs, milk, fruits and vegetables and a low consumption of plantain and was related to reduced odds of type 2 diabetes [17]. Moreover, previous analysis has revealed a strong association of increased triglycerides (≥1.695 mmol/L) with type 2 diabetes in this study population: multivariate adjusted OR 1.83 (95% CI: 1.13–2.97) [15]. Indeed, triglycerides were the main drivers of the association between the RRR-derived dietary pattern and type 2 diabetes in the present study. Clearly, as compared to factor analysis, the RRR method is an advance for the identification of diet-disease relationships. Both approaches are dimension reduction techniques which allow the calculation of dietary pattern scores. The previous factor analysis identified dietary patterns in this population that relied only on the combined consumption of foods and drinks. Beyond this, the RRR identified a dietary pattern that affects biomarker concentrations related to diabetes risk. From the RRR, we conclude that adherence to traditional food items and low preference for purchased foods relate to increased serum triglycerides—a strong risk factor for type 2 diabetes.

To our knowledge, the present study is the first study in SSA that identified a dietary pattern by using the RRR method. Overall, few epidemiological studies investigated the association between dietary patterns derived by RRR and type 2 diabetes. Although these studies used different intermediate markers as response variables including inflammatory biomarkers [12,14], HOMA-IR [13] and HbA1c, HDL-cholesterol, C-reactive protein (CRP) and adiponectin [11]; the explained variation in biomarkers of these studies is comparable to our findings.

To investigate the generalizability of RRR-derived dietary patterns, two studies validated the association of such patterns with diabetes risk in an independent European [22] and US population [23]. The European Prospective Investigation into Cancer and Nutrition (EPIC)-InterAct study found a good generalizability for three RRR dietary pattern scores based on the American Nurses’ Health Study (NHS), the German EPIC-Potsdam Study and the British Whitehall II Study [22]. The three dietary pattern scores were inversely associated with type 2 diabetes risk. In contrast, the American Framingham Offspring Study found a good generalizability for the NHS derived dietary pattern score, but the dietary pattern scores based on the European studies (EPIC-Potsdam Study and Whitehall II Study) were significantly less predictive for type 2 diabetes risk [23]. Thus the generalizability of dietary patterns associated with diabetes risk may be better in populations with comparable dietary intakes. Indeed, the transferability of previous RRR dietary patterns established in European and US populations to an urban Ghanaian population is complicated by the differences in the diet *per se*. However, all prospective studies found strong associations between dietary patterns obtained by RRR and type 2 diabetes. The strong relationships observed with the RRR method can partly be attributed to the use of disease-related biomarkers. In the Whitehall II Study, a diet high in low calories/soft drinks, onions, sugar-sweetened beverages, burgers and sausages, crisps and other snacks and white bread and low in medium-/high-fiber breakfast cereals, jam, French dressing/vinaigrette and whole meal bread was associated with a two- to threefold increase in diabetes risk by using HOMA-IR as the response variable [13]. The Insulin Resistance Atherosclerosis Study found a threefold to more than fourfold increased odds of diabetes associated with a dietary pattern high in red meat, low-fiber bread and cereal, dried beans, fried potatoes, tomato vegetables, eggs, cheese, and cottage cheese and low wine by using the inflammatory markers plasminogen activator inhibitor-1 and fibrinogen as the response variable [12]. In the NHS, Schulze *et al.* observed a pattern, which was high in sugar-sweetened soft drinks, refined grains, diet soft drinks and processed meat but low in wine, coffee, cruciferous vegetables, and yellow vegetable; this was related to a two- to threefold increase in diabetes risk by using inflammatory markers as the response variables [14]. Heidemann *et al.* found a dietary pattern among Caucasians, which was positively associated with HDL-cholesterol and adiponectin and inversely with HbA1c and CRP in the EPIC-Potsdam Study. This pattern was characterized by a high intake of fresh fruits and low intake of high-caloric soft drinks, beer, red meat, poultry, processed meat, legumes and bread (except wholegrain bread) and was inversely associated with the risk of type 2 diabetes [11].

An important difference between our observed dietary pattern and dietary patterns derived among Western populations is the inverse association of red meat with type 2 diabetes in our study population. Although not all previous meta-analyses found a positive association between red meat intake and type 2 diabetes [24], there is a large amount of epidemiologic evidence for this positive association among Western populations [25,26]. Possibly, the different types and preparation methods of red meat in urban Ghana compared to those among Western populations partially explain the inverse association with type 2 diabetes in our study. With regard to plantain—a major staple food in Ghana—we observed the highest contribution to the dietary pattern score and a positive association with type 2 diabetes. Plantain features a high glycemic index, and the content of simple sugars increases continuously during the ripening process [27]. Evidence from large epidemiologic studies showed that the glycemic index and the glycemic load are associated with a higher risk of type 2 diabetes [28]. Furthermore, frequent intake of carbohydrates has been related to an increase of fasting triglyceride concentrations and a reduction of HDL-cholesterol [29,30,31,32,33]. As for cassava and rice, it seemed contra-intuitive to us that its intake was positively associated with the pattern score, but inversely with type 2 diabetes. However, an inverse association between cassava flour and incident diabetes was also observed in a Brazilian study [34]. In our study population, plantain, cassava and rice were frequently consumed and the preparation methods were diverse including cooking, frying and pounding. Lacking a plausible biological explanation, we are speculating whether novel methods for the preparation of the traditional foods could explain the different associations of plantain and cassava with type 2 diabetes. Alternatively, the combined consumption of cassava and rice with other food groups [17] may represent dietary diversity, which is inversely associated with biomarkers of type 2 diabetes [35].

Notably, the biomarkers subjected to RRR in our study are similar to those in studies among Caucasian populations. However, the selection of biomarkers was limited to adiponectin, HDL-cholesterol and triglycerides based on established associations for type 2 diabetes. Inflammatory markers, such as CRP were not considered as response variables in our analysis due to the high prevalence of infectious diseases, complicating the interpretation of CRP as a risk factor for diabetes. Indeed, 13% of our study population had a *Plasmodium falciparum* infection [36]. Also, HOMA-IR was disregarded as a response variable, because of its metabolic proximity to type 2 diabetes. HOMA-IR is useful to determine pre-diabetic stages, but is not on the causal pathway for type 2 diabetes.

To the best of our knowledge, this study contributes uniquely to the literature; we are the first to identify a dietary pattern derived by RRR in an African population. We are aware that the RRR method is limited to existing studies with biomarkers or intermediate variables and knowledge of the diet–disease association. Nevertheless, this innovative method has the advantage to investigate the pathway between diet and disease as opposed to the exploratory factor analysis and cluster analysis which are entirely data driven. Given the limited data from SSA, a case-control design is useful to establish hypotheses on the relationship between dietary patterns and type 2 diabetes. However, these hypotheses require verification in prospective studies building a clear temporal relationship between dietary patterns and type 2 diabetes. Therefore, we cannot exclude that reverse causation contributes to our findings. Primarily, long-lasting type 2 diabetes might lead to changes of dietary behavior. Yet, at the time of study conduct, nutritional counseling was not part of the routine diabetes management at the hospital and thus, does not apply to our study population. Secondly, the concentrations of selected biomarkers chosen as response variables in the RRR might have changed during the course of diabetes. In this regard, we assessed whether the positive association of the dietary pattern score remained with FPG concentrations and HOMA-IR in the apparently healthy control group. Indeed, we found a nominal linear trend for FPG concentrations, while this was less clear for HOMA-IR. Also, the method of internal validation by random split technique confirmed the transferability and diabetes-association of the identified dietary pattern for urban Ghana. As for the dietary assessment, we admit that the retrospective FFQ bears the risk of under- or over-reporting of food items. The application of a locally specific FFQ by trained nurses of the same cultural background and language helped to keep these information biases to a minimum. Just since 2012, a national policy for the prevention and control of chronic non-communicable diseases exists in Ghana, including health promotions for a healthy diet [37]. Thus, public knowledge about the associations between specific food items and type 2 diabetes may not have been pronounced in the study area at the time of study conduct. Therefore, recall bias in reporting of specific foods seems unlikely in this population. Also, participants were not aware of their biomarker values at the time of dietary assessment. Even though we have adjusted for a large variety of known diabetes risk factors, we cannot rule out that unmeasured factors have influenced our observed results. Clearly, the hospital-based selection of controls helped to make the comparison groups more similar in terms of potential confounders, which are difficult to measure, such as socioeconomic background. Nevertheless, the hospital-based design leads to a study population that is not fully representative of the general one, and thus, the generalizability of our findings may be questionable.

## 5. Conclusions

In conclusion, we identified a dietary pattern that showed adherence to traditional food items and low preference for purchase foods in an urban Ghanaian population. This pattern was related to higher serum triglyceride concentrations and increased the risk of type 2 diabetes in this population.

## Supplementary Files

Supplementary File 1
