# Supplementary material for: A Dietary Pattern Derived by Reduced Rank Regression is Associated with Type 2 Diabetes in An Urban Ghanaian Population
Source: Nutrients. 2015 Jul 7;7(7):5497–514. doi: 10.3390/nu7075233 (PMC4517010; doi:10.3390/nu7075233)
Supplement: Supplementary File 1 [file nutrients-07-05233-s001.docx]

**Supplementary Information**

**Table S1.** Predictor variables for the reduced rank regression.

| **Original Food Group** | **Original Food Item** | **Predictor Variable for RRR** | **Scientific Rationale** |
| --- | --- | --- | --- |
| Starchy roots and tubers | Cassava | Cassava |  |
|  | Plantain | Plantain |  |
|  | Cocoyam | Cocoyam |  |
|  | Yam | Yam |  |
|  | Sweet potato | Sweet potato |  |
| Cereal and cereal products | Maize (Banku) | Maize (Banku) |  |
|  | Millet | Millet |  |
|  | Oats (porridge) | Oats (porridge) |  |
|  | Rice | Rice |  |
|  | Bread | Bread |  |
| Animal products | Fish | Fish |  |
|  | Red meat | Red meat |  |
|  | Poultry | Poultry |  |
|  | Eggs | Eggs |  |
|  | Milk | Milk |  |
|  | Crab | Crab |  |
| Legumes, nuts and beans | Beans | Beans |  |
|  | Groundnut | Groundnut |  |
|  | Agushie (pumpkin seeds) | Agushie (pumpkin seeds) |  |
| Fruits | Orange | Fruits | Single fruit items were combined into one food group “Fruits” to avoid overrepresentation of fruit intake |
|  | Mango |  |  |
|  | Papaya |  |  |
|  | Pineapple |  |  |
|  | Banana |  |  |
|  | Pae (avocado) |  |  |

**Table S1.** *Cont*.

| **Original Food Group** | **Original Food Item** | **Predictor Variable for RRR** | **Scientific Rationale** |
| --- | --- | --- | --- |
| Vegetables | Tomatoes | - | Excluded, because 100% of the participants daily consumed this item and thus did not contribute to variation in the usual diet |
|  | Sweet pepper | - | Excluded, because 100% of the participants daily consumed this item and thus did not contribute to variation in the usual diet |
|  | Garden egg | Garden egg |  |
|  | Okra | Okra |  |
|  | Green leafy vegetables | Green leafy vegetables |  |
|  | Carrot | Carrot |  |
|  | Cucumber | Cucumber |  |
|  | Lettuce | Lettuce |  |
| Fats and oils | Palm oil | Palm oil |  |
|  | Vegetable oil | Vegetable oil |  |
|  | Margarine | Margarine |  |
| Salt and spices | Salt | - | Excluded, because these items did not contribute to energy and macronutrient intake |
|  | Salt with iodine | - |  |
|  | Red pepper (dried) | - |  |
|  | Sugar | - |  |
| Sweets | Chocolate | Sweets | Single sweets were combined into one food group “Sweets”, because 89% of the participants consumed these items less than once a week |
|  | Ice cream |  |  |
|  | Toffee |  |  |
| Liquids | Water | - | Excluded, because 100% of the participants daily consumed this item; this item did not contribute to energy and macronutrient intake |

**Table S1.** *Cont*.

| **Original Food Group** | **Original Food Item** | **Predictor Variable for RRR** | **Scientific Rationale** |
| --- | --- | --- | --- |
|  | Juice | Juice |  |
|  | Soft drinks | Soft drinks |  |
|  | Coffee | Coffee |  |
|  | Milo (Hot chocolate) | Milo (Hot chocolate) |  |
|  | Beer |  | Beer, wine and spirits were combined into one food group “Alcoholic drinks”, because >90% of the participants never consumed these items |
|  | Wine | Alcoholic drinks |  |
|  | Spirits |  |  |

**Table S2.** Characteristics and biomarkers by quintiles of dietary pattern score among 325 controls in the training set.

| **Characteristics** | **Quintile of Dietary Pattern Score** | | | | |  |
| --- | --- | --- | --- | --- | --- | --- |
|  | **1** | **2** | **3** | **4** | **5** | ***p* for Trend** |
| n | 65 | 65 | 65 | 65 | 65 |  |
| Sex (female) | 50 (76.9) | 52 (80.0) | 52 (80.0) | 48 (74.0) | 53 (81.5) | 0.83 |
| Age (years) | 35.1 ± 14.1 | 44.8 ± 15.7 | 50.8 ± 13.3 | 53.6 ± 11.7 | 56.9 ± 12.6 | <0.001 |
| BMI (kg/m²) | 23.8 ± 4.5 | 26.2 ± 5.5 | 25.8 ± 4.8 | 26.6 ± 5.4 | 25.3 ± 4.3 | 0.09 |
| WHR | 0.82 ± 0.07 | 0.86 ± 0.07 | 0.88 ± 0.07 | 0.89 ± 0.06 | 0.88 ± 0.06 | <0.001 |
| very low SES | 7 (10.8) | 8 (12.3) | 13 (20.0) | 13 (20.0) | 24 (36.9) | 0.002 |
| Family history of diabetes | 15 (23.1) | 17 (26.2) | 16 (24.6) | 16 (24.6) | 12 (18.5) | 0.87 |
| Smoking (ever) | 2 (3.1) | 3 (4.6) | 1 (1.5) | 3 (4.6) | 2 (3.1) | 0.86 |
| Energy expenditure (kcal/d) | 1019 (860–1456) | 1261 (958–1524) | 1159 (793–1603) | 1349 (755–1724) | 1263 (743–1682) | 0.35 |
| Biomarkers |  |  |  |  |  |  |
| Adiponectin (mg/mL) | 9.23 (6.95–11.47) | 7.54 (5.67–10.61) | 8.83 (6.66–11.30) | 9.21 (7.27–10.81) | 8.56 (7.00–12.21) | 0.21 |
| HDL-cholesterol (mmol/L) | 1.52 (1.15–1.69) | 1.35 (1.06–1.55) | 1.36 (1.11–1.65) | 1.37 (1.19–1.58) | 1.28 (1.02–1.49) | 0.02 |
| Triglycerides (mmol/L) | 0.91 (0.74–1.23) | 1.13 (0.86–1.51) | 1.15 (0.89–1.73) | 1.27 (1.02–1.84) | 1.40 (0.99–1.73) | <0.001 |

**Table S2.** *Cont*.

| **Characteristics** | **Quintile of Dietary Pattern Score** | | | | |  |
| --- | --- | --- | --- | --- | --- | --- |
|  | **1** | **2** | **3** | **4** | **5** | ***p* for Trend** |
| Food intake (servings/week) ^1^ | | |  |  |  |  |
| positive association | |  |  |  |  |  |
| Plantain | 1.5 (0.5–3.5) | 3.5 (1.5–5.5) | 3.5 (1.5–7.0) | 5.5 (1.5–7.0) | 7.0 (5.5–7.0) | <0.001 |
| Cassava | 1.5 (0.5–3.5) | 1.5 (1.5–3.5) | 1.5 (1.5–3.5) | 3.5 (1.5–5.5) | 7.0 (3.5–7.0) | <0.001 |
| Garden egg | 3.5 (1.5–7.0) | 3.5 (1.5–7.0) | 5.5 (3.5–7.0) | 7.0 (3.5–7.0) | 7.0 (7.0–7.0) | <0.001 |
| inverse association | |  |  |  |  |  |
| Rice | 7.0 (5.5–7.0) | 5.5 (3.5–7.0) | 3.5 (1.5–5.5) | 3.5 (1.5–7.0) | 1.5 (0.5–3.5) | <0.001 |
| Juice | 1.5 (0.5–3.5) | 0.5 (0–1.5) | 0.5 (0–1.5) | 0 (0–0.5) | 0 (0–0.5) | <0.001 |
| Vegetable oil | 3.5 (3.5–7.0) | 3.5 (1.5–5.5) | 3.5 (1.5–3.5) | 1.5 (0.5–3.5) | 1.5 (0.5–3.5) | <0.001 |
| Eggs | 1.5 (0.5–3.5) | 0.5 (0.5–1.5) | 0.5 (0.5–1.5) | 0.5 (0–0.5) | 0.5 (0–0.5) | <0.001 |
| Milo (chocolate drink) | 1.5 (1.5–5.5) | 1.5 (0.5–3.5) | 1.5 (0.5–3.5) | 0.5 (0–1.5) | 0.5 (0–1.5) | <0.001 |
| Sweets | 0.5 (0.5–1.5) | 0.5 (0–0.5) | 0.5 (0–0.5) | 0 (0–0.5) | 0 (0–0.5) | <0.001 |
| Red meat | 3.5 (1.5–7.0) | 1.5 (0.5–5.5) | 1.5 (0.5–1.5) | 0.5 (0.5–1.5) | 0.5 (0.5–1.5) | <0.001 |

Values are expressed as mean ± standard deviation, participant number (%) or median (interquartile range) ^1^ We included 35 food items in our analysis, the ten food items that loaded highest on the dietary pattern derived by reduced rank regression are presented here.


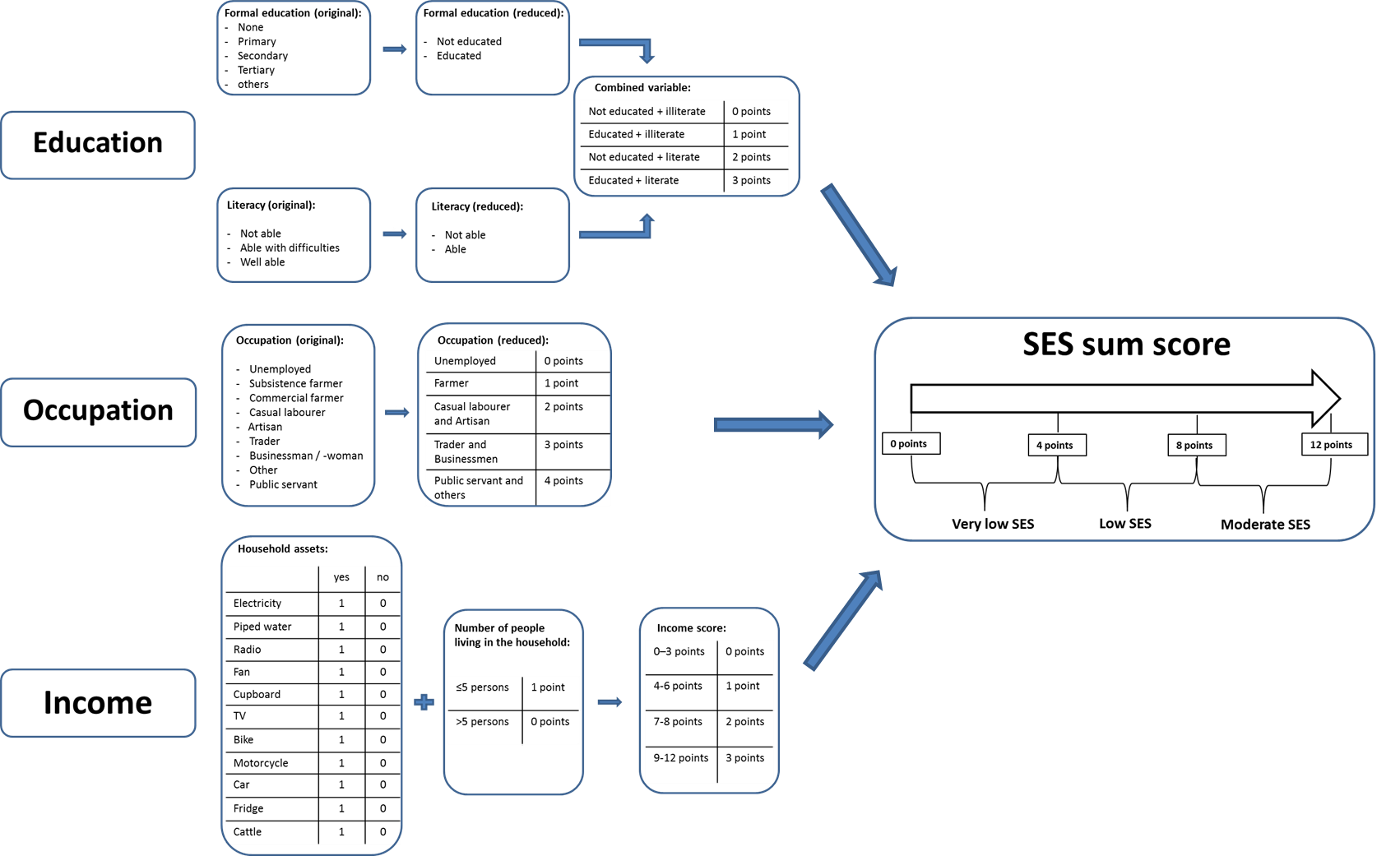


**Figure S1.** Construction of the socio-economic (SES) sum score. The common proxy markers education, occupation and income were used to construct a SES sum score ranging from 0 to 12 points. First a new variable was constructed by combining the information regarding education and literacy. This new variable with four characteristics covered information about having a graduation and being able to write and read, points from 0 to 3 were given. Occupation, originally a variable with nine characteristics, was condensed to a new variable with five characteristics, given the points 0 to 4. Due to the problems of a valid ascertainment of income in Ghana, a list of 11 household assets was recorded. An income score ranging from 0 to 12 points was constructed based on these assets and the number of people living in the household. The income score was divided into quartiles, given the points 0 to 3. To create the overall SES sum score the points of education, occupation and the income score were summed up to a score ranging from 0 to 12 points.

**Figure S2.** Directed acyclic graph of the reduced rank regression. In reduced rank regression, a response score is established by maximizing the variation in biomarkers (adiponectin, triglycerides, HDL-cholesterol) explained by the predictor variables (food groups). The dashed lines indicate the well-established causal relationship between biomarkers and type 2 diabetes. The biomarkers score is then projected onto the surface of food groups to determine a dietary score, which is then subjected as an exposure variable to the logistic regression with the outcome type 2 diabetes

© 2015 by the authors; licensee MDPI, Basel, Switzerland. This article is an open access article distributed under the terms and conditions of the Creative Commons Attribution license (http://creativecommons.org/licenses/by/4.0/).
